# Distinct manifold encoding of navigational information in the subiculum and hippocampus

**DOI:** 10.1126/sciadv.adi4471

**Published:** 2024-01-31

**Authors:** Shinya Nakai, Takuma Kitanishi, Kenji Mizuseki

**Affiliations:** ^1^ Department of Physiology, Graduate School of Medicine, Osaka Metropolitan University, Osaka 545-8585, Japan.; ^2^ Department of Physiology, Graduate School of Medicine, Osaka City University, Osaka 545-8585, Japan.; ^3^ Department of Life Sciences, Graduate School of Arts and Sciences, The University of Tokyo, Meguro, Tokyo 153-8902, Japan.; ^4^ Komaba Institute for Science, The University of Tokyo, Meguro, Tokyo 153-8902, Japan.; ^5^ PRESTO, Japan Science and Technology Agency (JST), Kawaguchi, Saitama 332-0012, Japan.

## Abstract

The subiculum (SUB) plays a crucial role in spatial navigation and encodes navigational information differently from the hippocampal CA1 area. However, the representation of subicular population activity remains unknown. Here, we investigated the neuronal population activity recorded extracellularly from the CA1 and SUB of rats performing T-maze and open-field tasks. The trajectory of population activity in both areas was confined to low-dimensional neural manifolds homoeomorphic to external space. The manifolds conveyed position, speed, and future path information with higher decoding accuracy in the SUB than in the CA1. The manifolds exhibited common geometry across rats and regions for the CA1 and SUB and between tasks in the SUB. During post-task ripples in slow-wave sleep, population activity represented reward locations/events more frequently in the SUB than in CA1. Thus, the CA1 and SUB encode information distinctly into the neural manifolds that underlie navigational information processing during wakefulness and sleep.

## Teaser

Subiculum manifolds involve accurate navigational information, common geometry, and ripple-associated reward representation.

## INTRODUCTION

Spatial navigation is essential for survival. Information supporting spatial navigation is expressed in terms of position, speed, and head direction (*1*–*3*). The hippocampal formation, consisting of the dentate gyrus, CA3, CA2, CA1, and subiculum (SUB), plays an essential role in processing navigational information (*4*). This processing occurs at different levels of neural activity, including single-neuron activity (*1*), pairwise neuron activity (*5*), and neuronal population activity, which is often represented as neural manifolds (*6*). Information conveyed by the hippocampus has been extensively studied and is involved in spatial navigation and memory (*7*–*9*). However, the SUB had been investigated much less until recently, and its neuronal representation, particularly as a population, remains poorly understood.

The anatomical connections between the SUB and other regions suggest that the SUB plays an important role in navigational information processing (*10*, *11*). The SUB receives afferent projections from the brain regions conveying spatial information, including the hippocampal CA1 area, entorhinal cortex, retrosplenial cortex, and nucleus reuniens (*12*, *13*). This information can be integrated via dense recurrent connections in the SUB (*14*) and sent to various regions, including the retrosplenial cortex, nucleus accumbens, medial mammillary body, and anteroventral thalamic nucleus (*15*–*19*). These downstream areas are involved in spatial memory, reward processing, and emotional systems, suggesting that the SUB acts as a functional hub by providing navigational information (*20*–*22*). Loss-of-function studies have shown that the SUB serves as a unique computational unit (*23*–*25*).

The SUB contains neurons representing diverse navigational information, including position, speed, movement axes, reward, and task structure (*26*–*32*). In addition, there are cells representing the future choice of path or boundary/landmarks in the environment (*31*, *33*, *34*). Such spatial representation is reminiscent of the CA1 area, containing cells representing position, speed, and future choice of the path (*1*, *2*, *35*). However, the coding strategies of CA1 and SUB neurons differ substantially (*11*, *27*). SUB neurons generally have low selectivity and multiple receptive fields (*26*, *27*, *36*, *37*) but exhibit high firing rates and mixed selectivity (*31*, *32*, *37*). These features contribute to the noise-resistant and efficient information transfer from the SUB to its downstream regions (*31*, *32*, *37*). In addition, during sharp-wave ripples (SWRs), SUB neurons accurately transmit information in a projection-specific manner (*31*). In contrast to such representations at the single-neuron level, data for this at the population level remain limited. The spike times of a given neuron are more precisely predicted using spike times of simultaneously recorded neurons in addition to the features of the receptive field of that neuron, input from the external environment, and the animal's behavior (*38*, *39*). Therefore, exploring neuronal population dynamics is essential to understanding information processing in the brain.

The time series of neuronal population activity can be described as a trajectory in a high-dimensional space in which the instantaneous activity of individual neurons is represented by their respective coordinate axes. Owing to the neuronal network and its input properties, a high degree of correlation and redundancy exists between the activities of individual neurons (*40*, *41*); hence, potential trajectories of population activity are often confined to a subregion of this space: a neural manifold embedded within the high-dimensional space (*42*, *43*). Neural manifolds have been identified in various systems, including head direction cells in the anterodorsal thalamic nucleus (*44*), value-coding cells in the retrosplenial cortex (*45*), grid cells in the entorhinal cortex (*46*), and place cells in the CA1 (*47*–*49*). Neural manifolds in the CA1 of mice performing a linear-track task in a real environment (*47*) and an accumulating-tower task in a virtual space (*48*) represent physical and abstract variables relevant to the task, respectively. The information representation of single neurons in the SUB and CA1 has similarities and differences; however, the representation of the SUB population activity remains unknown. Because the neural manifold reflects not only the tuning of single neurons but also higher-order coactive relationships between neurons, the neural manifold does not necessarily inherit a specific neuronal tuning (*50*–*52*). Therefore, similarities and differences in neuronal manifolds between the SUB and CA1 cannot be inferred from the similarities and differences in representations of individual neurons between the SUB and CA1. We investigated the neuronal population activity recorded from the CA1 and SUB of rats performing T-maze and open-field tasks. Here, we found that the low-dimensional neural manifolds formed by the SUB encode various types of navigational information more accurately than the CA1 and exhibit common structures across rats and tasks, providing the basis for information processing in spatial navigation.

## RESULTS

### CA1 and SUB form low-dimensional neural manifolds during spatial tasks

We used large-scale recoding data obtained via 256-channel silicon probes to monitor neuronal firing from the CA1 and SUB in rats performing the T-maze and open-field tasks (*31*). For the T-maze task, we analyzed 302 and 282 putative principal neurons in the CA1 and SUB from 13 and 15 recording sessions in eight and nine rats, respectively. For the open-field task, we analyzed 265 and 282 putative principal neurons in the CA1 and SUB from 10 and 15 recording sessions in six and nine rats, respectively.

**Fig. 1.**
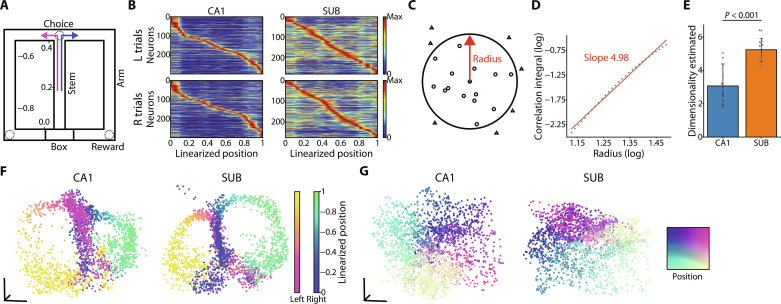
Low-dimensional neural manifold during the T-maze and open-field tasks in the CA1 and SUB. (**A**) Schematic illustration of the T-maze task. The numbers indicate linearized positions along the maze. (**B**) Rate maps of all CA1 (left) and SUB (right) neurons, excluding silent neurons (average firing rate is 0 Hz) on the left and right trials. The maps were normalized by peak firing rates and sorted by the position showing the peak firing rate. Left [top panels (L)] and right [bottom panels (R)] trials are shown separately. (**C**) Schematic illustration of the GP algorithm used to estimate dimensionality. Points inside and outside the hypersphere are indicated by circles and triangles, respectively. (**D**) Representative estimation of the latent dimensionality of population activity during the T-maze task embedded in a high-dimensional space. The slope of the radius *r* versus the correlation integral *C*(*r*) plotted on a log-log scale corresponds to dimensionality. The red line indicates the slope obtained using the least squares method. (**E**) Dimensionality estimates during the T-maze task in the CA1 and SUB. *P* < 0.001, Welch’s *t* test. (**F**) Low-dimensional neural manifolds during a single T-maze task session in the CA1 (left) and SUB (right) visualized using Isomap in a three-dimensional space. Single points correspond to the neuronal population activity at each time bin [same for (**G**)]. The plots are color-coded according to the rat position in the T-maze, distinguishing between right-handed (cool colors) and left-handed (warm colors) turns. Color bars show the linearized positions. (G) Same as (F) but for the open-field task. The plots are color-coded according to the rat position in the open field.

The time series of neuronal population activity can be captured as point clouds in a high-dimensional space with the firing activity of each neuron as the axis. This neuronal activity is often restricted to a subregion of this high-dimensional space: a low-dimensional neural manifold (*42*–*49*). Therefore, we first examined the latent dimensions of neuronal population activity in the CA1 and SUB of rats performing the T-maze task (Fig. 1, A and B). For this, we used the Grassberger-Procaccia (GP) algorithm (*47*, *48*, *53*), which estimates the dimensions of the point cloud based on a function that accumulates the number of neighboring points in a hypersphere of a certain radius (Fig. 1C). This revealed that the activity of the neuronal population consisting of dozens of neurons was represented in approximately three and five dimensions in the CA1 and SUB, respectively (Fig. 1, D and E; CA1, 3.07 ± 1.32; and SUB, 5.24 ± 0.70; means ± SD). The dimensions were significantly higher in the SUB than in the CA1 (Fig. 1E). One of the characteristics of SUB principal neurons is their high firing rate (*26*, *31*). To remove the effects of the dynamic range of firing rates, we estimated the dimensionality using *z*-scored firing rates instead of firing rates. We observed no change in the dimensionality and consistent differences between the regions (CA1, 3.01 ± 1.30; and SUB, 5.73 ± 0.87). Similar dimensionality values were observed in the open-field task (CA1, 3.61 ± 1.10; and SUB, 5.84 ± 0.79).

We visualized low-dimensional neural manifolds using the Isomap method, one of the nonlinear dimensionality reduction methods, which potentially extracts low-dimensional structures embedded in higher-dimensional spaces. This analysis revealed that the trajectory of population activity during the T-maze task in three-dimensional space displayed a figure-8 structure (Fig. 1F). In addition, the population activity during the open-field task displayed a square-shaped structure (Fig. 1G), indicating a similarity to the external environment. Hereafter, we analyzed population activity during the T-maze task unless otherwise specified.

### Structural features of the low-dimensional neural manifold

**Fig. 2.**
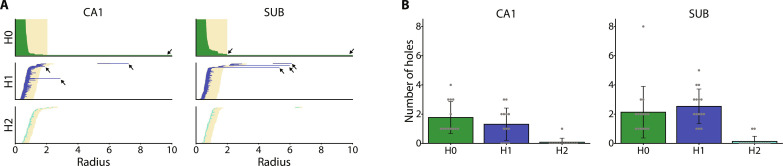
Topological feature analysis using persistent homology. (**A**) Barcode plots indicate topological features of the neural manifold in three-dimensional space during a single T-maze session. The *x* axis shows the radius of the sphere placed at individual data points. The start and end radii of each bar indicate the appearance and disappearance of holes, respectively. CA1 (left) and SUB (right) are shown separately. H0, H1, and H2 indicate holes with zero, one, and two dimensions, respectively, where holes with lifetime lengths ≥ 95th, ≥ 85th, and ≥ 0th percentiles are plotted. Orange shading indicates the 99.9th percentile of the null distribution of hole lifetimes. Arrows indicate bars longer than the null distribution (i.e., robust holes). (**B**) Number of robust holes in each dimension (H0, H1, and H2) of the CA1 (left) and SUB (right).

We characterized the structural features of the low-dimensional neural manifold during the T-maze task visualized via the Isomap method using persistent homology (*54*, *55*) and D2 shape distribution (*56*). Persistent homology, often used in the analysis of neural manifolds, extracts the topological features by finding holes of various dimensions on a point cloud (*44*, *46*). Using persistent homology, we generated barcode plots for low-dimensional neural manifolds represented in a three-dimensional space (Fig. 2A). The barcode plots show the lifetimes of the holes, with holes with 0, 1, and 2 dimensions corresponding to the components, rings, and cavities, respectively. Null distributions of hole lifetimes were generated by randomly and circularly shifting the spike train of individual neurons independently. The holes with lifetimes longer than the 99.9th percentile of the null distributions were considered robust (Fig. 2A). The neural manifolds in the CA1 and SUB had approximately two to three ring structures on average across all sessions (Fig. 2B), which is consistent with the shape of the figure-8 maze (Fig. 1F).

**Fig. 3.**
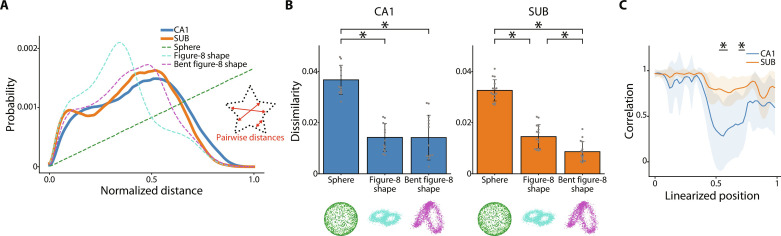
Geometric feature analysis using D2 shape distribution. (**A**) Representative D2 shape distribution plots for three-dimensional neural manifolds obtained from a single T-maze session in the CA1 (blue) and SUB (orange). The *x* axis indicates the normalized distance between the points, while the *y* axis shows the probability of that distance. The colored dotted lines (green, cyan, and magenta) represent the respective feature models (sphere, figure-8 shape, and bent figure-8 shape, respectively). A schematic diagram of the pairwise distances is shown in the inset. (**B**) Dissimilarity between the neural manifolds of CA1 (left), SUB (right), and each feature model. CA1, *F*_2,36_ = 47.12, *P* < 0.001; and SUB, *F*_2,42_ = 128.5, *P* < 0.001; one-way ANOVA; *P < 0.05, Bonferroni test. (**C**) Population vector (PV) correlation between left and right trials for CA1 (blue) and SUB (orange) neurons (lines and shaded areas, means ± SD). *P < 0.05, Welch’s *t* test with Bonferroni correction.

The D2 shape distribution represents the shape signatures of structures, and the dissimilarity of the structures can be evaluated by comparing shape distributions (*56*). First, we generated the D2 shape distribution by calculating the distances of all combinations of points on the neural manifold embedded in a three-dimensional space (Fig. 3A). Next, we generated feature models and examined their D2 shape distribution. The sum of the absolute probability difference between the data and model was quantified to estimate the dissimilarity. The D2 shape distribution appeared bimodal (Fig. 3A), with similar bimodality coefficients between the CA1 and SUB (CA1, 0.48 ± 0.051; and SUB, 0.45 ± 0.041; *P* = 0.11, Welch’s *t* test). The shape of the neural manifold during the T-maze task was similar to the figure-8 shape in both the CA1 and SUB (Fig. 3B). In particular, the shape of the neural manifold in the SUB was more similar to the bent figure-8 shape than to the planar figure-8 shape (Fig. 3B). Within the bent figure-8 shape, the distances between points on the manifold for the left and right arms/reward zones can be small. Consistent with this, the correlations between left- and right-turn trial population vectors (PVs) of SUB neurons were higher than those of CA1 neurons at the arms locations (Fig. 3C). Moreover, the SUB neurons showed higher spatial tuning correlation between the left and right arms/reward zones than CA1 neurons (CA1, 0.26 ± 0.090; and SUB, 0.41 ± 0.13; *P* = 0.0016 by Welch’s *t* test). These results show that the neuronal population activity in the CA1 and SUB forms a low-dimensional neural manifold that is homoeomorphic to the external task environment. The difference in the geometry of neural manifolds between the CA1 and SUB suggests that the encoding of information to the neural manifolds differs between the regions.

### The low-dimensional neural manifold represents diverse navigational information

As CA1 and SUB neurons represent various types of navigational information (*11*), we examined whether the neural manifold in these regions conveyed such navigational information. The neural manifold reflects the activity of both individual neurons and neuronal ensembles. Therefore, even if some individual neurons in the CA1 and SUB encode position, speed, and path, it is not obvious whether these types of information are encoded in the CA1 and SUB manifolds, which are extracted from population activities in an unsupervised manner without any information on the animal’s behavior.

**Fig. 4.**
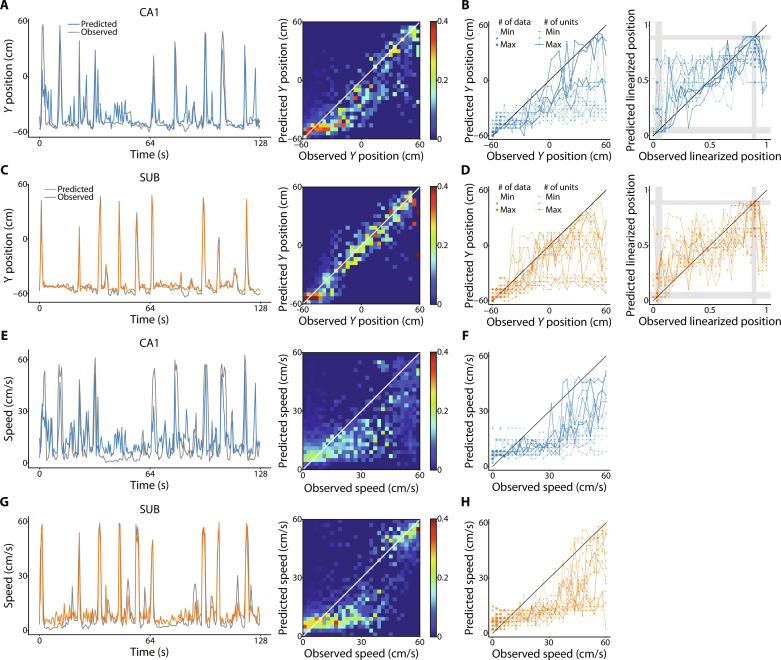
Position and speed information represented by three-dimensional neural manifolds. (**A**) Decoding the rat position from the neural manifold over time in the CA1. The gray and colored lines indicate the observed and predicted rat positions, respectively (left). The heatmap shows the joint probability of the observed and predicted rat positions for a single session (right). For visualization, only the *Y* position is shown. (**B**) The maximum joint probability of the observed and predicted rat positions for all sessions is shown for the *Y* position (left) and linearized position (right). The gray shaded areas indicate the locations of the start box (0.02 to 0.08) and reward areas (0.86 to 0.90). The dot size and line width represent the number of data points and the number of neurons used for constructing manifolds, respectively. (**C**) Same as (A) but for the SUB. (**D**) Same as (B) but for the SUB. (**E**) Same as (A) but for the decoding of running speed. (**F**) Same as (left B) but for the decoding of running speed. (**G**) Same as (E) but for the SUB. (**H**) Same as (F) but for the SUB.

We used Gaussian process regression (GPR) analysis (*57*) to decode the position of rats from the coordinates on the neural manifold in the three-dimensional space (*48*) and performed twofold cross-validation, in which data in each session were randomly sorted into two groups with the same size; each group was used as test data, while the remaining group was used as training data. The results showed that the rat’s position during the T-maze task was successfully decoded from the neural manifold (Fig. 4, A to D). The speed of the movement during the T-maze task was also decodable (Fig. 4, E to H). When we performed the decoding from the nine-dimensional neural manifold, the noisiness of the decoding plots (Fig. 4, B, D, F, and H; measured as the SD of the difference between the predicted variable and the diagonal) decreased for both position [CA1: 5.62 ± 1.76 versus 5.09 ± 1.95 (three versus nine dimensions), *P* = 0.0047 by paired *t* test; and SUB: 5.65 ± 1.81 versus 3.92 ± 1.86, *P* = 0.0020] and speed (CA1: 5.44 ± 1.31 versus 4.66 ± 1.70, *P* = 0.0024; and SUB: 4.86 ± 1.31 versus 3.54 ± 0.93, *P* < 0.001). Thus, the neural manifolds with three dimensions retain fundamental, decodable position and speed information, while the high-dimensional population activity is likely to contain fine navigational information.

**Fig. 5.**
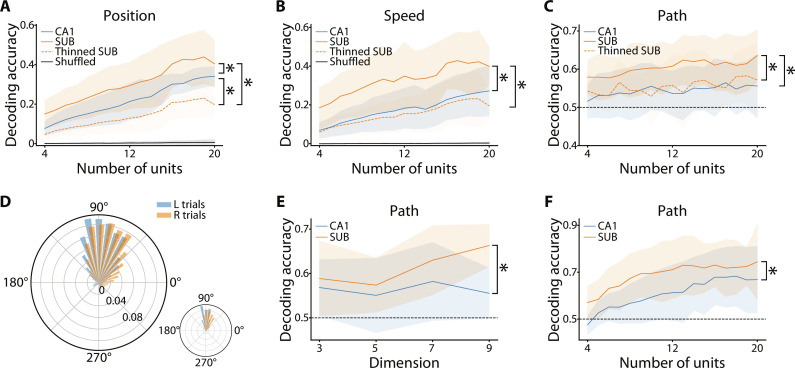
Diverse navigational information represented by low-dimensional neural manifolds. (**A**) Decoding accuracy of the position using three-dimensional neural manifolds for the CA1 (solid blue), SUB (solid orange), and firing rate-equated SUB by removing spikes (thinned SUB; dotted orange). The *x* axis indicates the number of neurons used to construct the neural manifold [the same for (**B**), (**C**), and (**F**)]. The black line shows the chance level estimated using the shuffling procedure [the same for (B)]. *F*_2,546_ = 173.42, *P* < 0.001, two-way ANOVA; *P < 0.001, post hoc Bonferroni test. (B) Same as (A) but for decoding accuracy of the speed. *F*_2,546_ = 139.59, *P* < 0.001, two-way ANOVA; *P < 0.001, post hoc Bonferroni test. (C) Decoding accuracy of the path using three-dimensional neural manifolds for the CA1 (blue), SUB (orange), and firing rate-equated SUB (thinned SUB; dotted orange). The black dotted line indicates the chance level [same as (**E**) and (F)]. *F*_2,546_ = 48.97, *P* < 0.001, two-way ANOVA; *P < 0.001, post hoc Bonferroni test. (**D**) Average distributions of the head direction in the start box for future left- and right-turn trials. *P* < 0.001, Watson *U*^2^ test. Inset: Distribution of a single session. (E) Decoding accuracy of the path for the CA1 (blue) and SUB (orange) after equating the distributions of head directions between left- and right-turn trials by randomly removing time bins associated with head-direction bins with excess probability over the other choice for each session. Dimension: Embedding dimension in the Isomap procedure. Twenty units were used for decoding. *F*_1,44_ = 6.15, *P < 0.05, two-way ANOVA. (F) Decoding accuracy of the path using pairwise coactivities in the CA1 (blue) and SUB (orange). *F*_1,354_ = 52.05, *P < 0.05, two-way ANOVA. Lines and shaded areas in (A) to (C), (E), and (F), means ± SD.

The decoding performance is influenced by the number of neurons included in the dataset (*31*). Therefore, we evaluated the decoding accuracy of position and speed by matching the number of neurons to generate the neural manifold. Our results indicated that the SUB manifold showed higher decoding accuracy for both position and speed than the CA1 manifold (Fig. 5, A and B). SUB neurons have higher mean firing rates than CA1 neurons (*26*, *31*). Therefore, we equated the mean firing rate of CA1 (1.43 Hz) and SUB (5.80 Hz) neurons by randomly removing 75% of the spikes from individual SUB neurons (thinned SUB in Fig. 5). Thinned SUB exhibited less accurate decoding than CA1 for position (Fig. 5A) and a decoding as inaccurate as that of the CA1 for speed (Fig. 5B). Thus, the higher firing rate contributes to the accurate decoding of information from the SUB manifold.

Next, we determined whether neural manifolds convey navigational information linked to spatial memory, such as future paths in the alternation task. Previous studies have identified neurons in the CA1 and SUB that represent future choices (*31*, *35*). Specifically, SUB neurons have higher decoding accuracy than CA1 neurons (*31*). We decoded the path (left or right arm) that the rats chose at the future T-junction using the coordinates on the manifold during their stay in the start box immediately preceding the choice of that path. Our results showed that the path was decodable from the neural manifold and that decoding accuracy was higher for the SUB than for the CA1 (Fig. 5C). The thinned SUB showed less accurate decoding than the SUB (Fig. 5C). The distributions of the head direction in the start box before left- and right-turn trials appeared similar but showed significant differences (Fig. 5D). This variability in the distributions may have potentially contributed to the observed path decoding result. Therefore, for each session, we equated the head-direction distributions in the box before constructing the neural manifold and decoding the future path. The resultant decoding accuracy in the SUB was only moderately better than that in the CA1 with an embedding dimension of three [*F*_1,61_ = 5.17, *P* = 0.027, two-way analysis of variance (ANOVA)]. However, the latent dimensionality of the SUB was larger than three (Fig. 1E); when higher embedding dimensions were included, the SUB showed a prominently greater decoding accuracy than the CA1 (Fig. 5E). Thus, the SUB manifold, although predominantly evident at higher dimensionality, carries a larger amount of decodable path information than the CA1 manifold, which cannot be attributed to the different head directions.

Both the activity of individual neurons and the coordinated activity between neurons, including pairwise coactivation, are involved in forming neural manifolds. The path choice could also be decoded using the pairwise coactivation between neurons in the start box (Fig. 5F), suggesting that navigational information is not solely represented by the activity of individual neurons but also by the coordinated activity between neurons. Moreover, the decoding accuracy of pairwise coactivation was higher for the SUB than for the CA1 (Fig. 5F). Together, these results indicate that the neural manifolds in the SUB represent various types of navigational information and achieve higher decoding accuracy than those in the CA1.

### Differences in decoding accuracy between CA1 and SUB are consistent irrespective of parameters

**Fig. 6.**
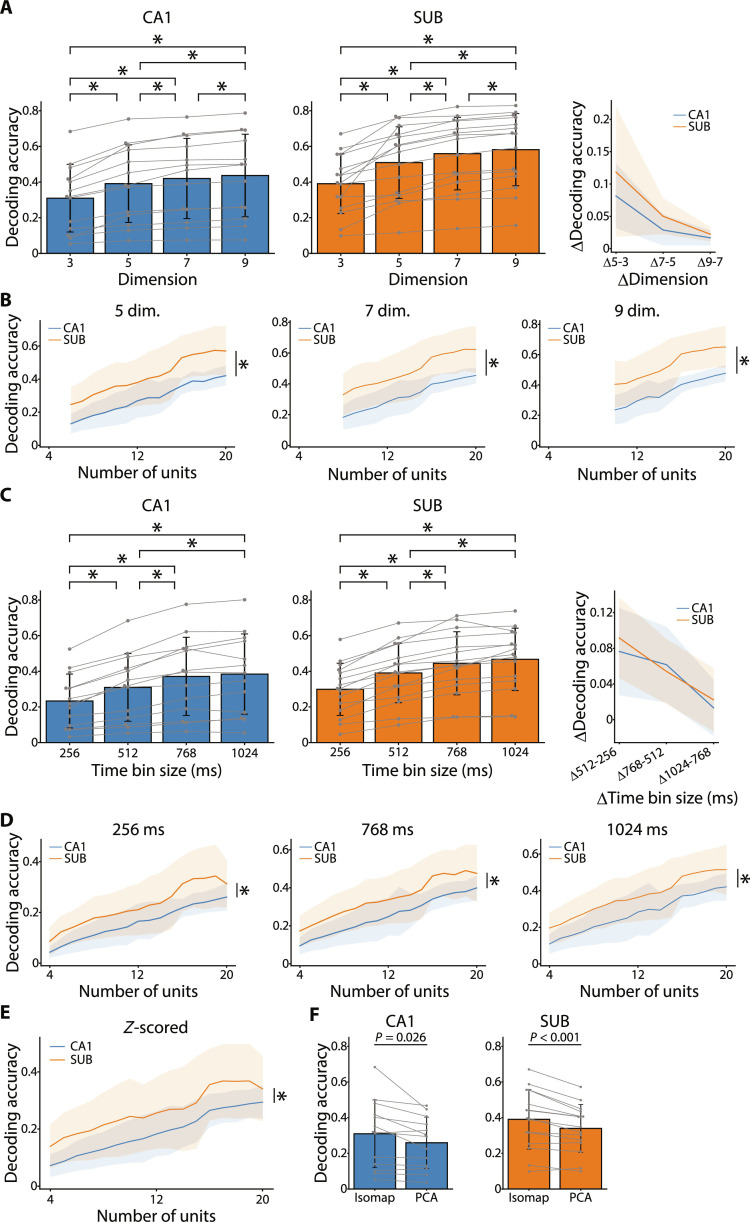
Consistency of the difference in position decoding accuracy between the CA1 and SUB. (**A**) Decoding accuracy of position for the CA1 and SUB [same for (**C**) and (**F**)] calculated from three-, five-, seven-, and nine-dimensional neural manifolds, respectively. *P < 0.05, paired *t* test with Bonferroni correction following one-way repeated-measures ANOVA. Right: The increase rate of decoding accuracy with dimensionality increases. The same data are plotted for dimension 3 (A), 512-ms bin size (C), and Isomap (F) for both the CA1 and SUB. (**B**) Decoding accuracy of position for the CA1 and SUB using neural manifolds embedded in five (left), seven (middle), and nine (right) dimensions (solid lines and shaded area, means ± SD). The *x* axis indicates the number of units used to construct the neural manifold [same for (D)(**D**) and (**E**)]. Left: region, *F*_1,302_ = 104.71, *P < 0.001; middle: region, *F*_1,250_ = 106.07, *P < 0.001; right: region, *F*_1,198_ = 100.51, *P < 0.001, two-way ANOVA. (C) Decoding accuracy calculated for three-dimensional neural manifolds constructed with 256-, 512-, 768-, and 1024-ms time bin sizes. *P < 0.05, paired *t* test with Bonferroni correction following one-way repeated-measures ANOVA. Right: The increase rate of decoding accuracy with time bin size increase. (D) Decoding accuracy using three-dimensional neural manifolds constructed with 256-ms (left), 768-ms (middle), and 1024-ms (right) time bin sizes. Left: region, *F*_1,354_ = 65.03, *P < 0.001; middle: region, *F*_1,354_ = 72.17, *P < 0.001; right: region, *F*_1,354_ = 70.40, *P < 0.001, two-way ANOVA. (E) Decoding accuracy using three-dimensional neural manifolds constructed with *z*-scored firing rates. Region, *F*_1,354_ = 54.73, *P < 0.001, two-way ANOVA. (F) Decoding accuracy using the three-dimensional neural manifold constructed by Isomap and PCA. CA1, *P* = 0.026; and SUB, *P* < 0.001, paired *t* test.

We investigated the consistency of the differences in decoding accuracy of the neural manifolds between the CA1 and SUB by varying the number of embedding dimensions of neuronal population activity and the size of time bins to decode the animal’s position. As for the number of embedding dimensions, we compared the decoding accuracy of the rat’s position with three, five, seven, and nine embedding dimensions. The decoding accuracy gradually improved as the number of embedding dimensions increased, and the improvement became smaller when approximately five dimensions were reached in both regions (Fig. 6A). A comparison of decoding accuracy with matching the number of neurons used to generate the neural manifold showed that the SUB had consistently higher decoding accuracy than the CA1 regardless of the dimension (Fig. 6B).

We also observed that, as the time bin size increased, the decoding accuracy improved, which was similar for both regions (Fig. 6C). Furthermore, a comparison of decoding accuracy with matching the number of neurons used to generate the neural manifold for each time bin size revealed that the SUB exhibited higher decoding accuracy than the CA1 regardless of time bin size, indicating the consistency of between-region differences (Fig. 6D).

To remove the effects of the dynamic range of firing rates on the decoding accuracy, we next generated neural manifolds using the *z*-scored firing rates of individual neurons and compared the decoding accuracy between regions. Again, the decoding accuracy was higher in the SUB than in the CA1 (Fig. 6E). These results indicate that the between-region differences in decoding accuracy are consistent irrespective of the analysis parameters and, therefore, would reflect distinct coding strategies between the CA1 and SUB.

Last, to examine the influence of dimensionality reduction methods on decoding accuracy, we compared the Isomap and principal component analysis, which are nonlinear and linear dimensionality reduction methods, respectively. Similar to previous findings on the anterodorsal thalamic nucleus (*44*), Isomap resulted in a more accurate decoding of the rat’s position for both the CA1 and SUB (Fig. 6F), suggesting that Isomap better extracts neural manifolds in the present experimental systems.

### Structural similarity of low-dimensional neural manifolds across rats

**Fig. 7.**
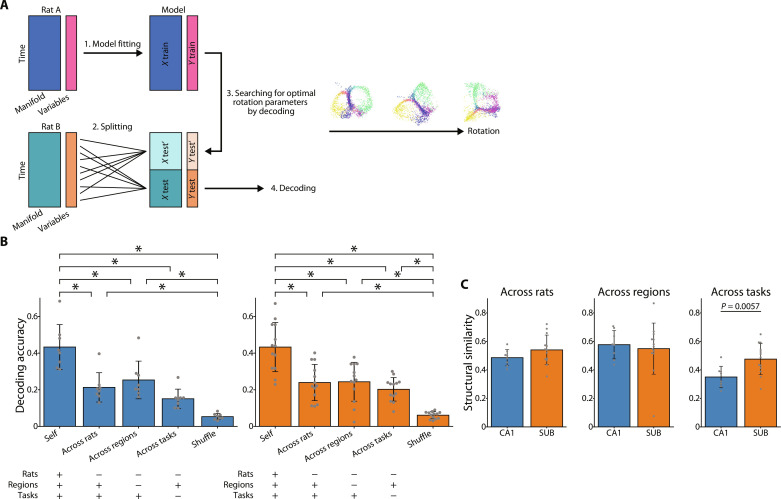
Structural similarity of three-dimensional neural manifolds across rats. (**A**) Schematic illustration of the decoding analysis across rats, regions, and tasks. A regression model trained on one rat (rat A) was used to decode the position of another rat (rat B) after aligning its neural manifold with optimal rotation parameters in a three-dimensional embedding space (see Materials and Methods). (**B**) The position decoding accuracy comparison in the CA1 (left, blue) and SUB (right, orange). For the decoding methods of “across rats,” “across regions,” and “across tasks,” a regression model trained on one rat was used to decode the position of another rat. For the across-rat method, the neural manifolds from the same brain region during the T-maze task were used for the model training and decoding. For the across-region method, the manifolds from different brain regions, but during the same T-maze task, were used for the model training and decoding. For the across-task method, the neural manifolds in the same region were used for the model training and decoding, the T-maze task was used for the decoding, and the open-field task was used for the model training. For the “self” method, half of the dataset from each rat during the T-maze task was used to train the model fitting, and the other half of the dataset from the same rat was used to decode the rat’s position. For the “shuffle” method, the circularly and randomly shifted spike trains were used for the model training. *P < 0.05, Bonferroni test following one-way ANOVA. In the table below, “+” or “−” indicate that the rat, brain region, or task used for the model training is the same or different from that used for the decoding, respectively. (**C**) Comparison of structural similarity. *P* = 0.0057, Welch’s *t* test.

Our results indicated that the neural manifolds in the CA1 and SUB are similar to the external environment. These results and those of previous studies suggest that the structure of the neural manifold is task-specific rather than individual-specific (*48*). Therefore, we determined whether the neural manifolds had a common structure across rats, regions (i.e., CA1 and SUB), and tasks (i.e., T-maze and open-field tasks). To this end, we first trained a regression model to predict the position from the neural manifold coordinates in one rat and then applied this model to decode another rat’s position after aligning its neural manifold with optimal rotation parameters in a three-dimensional embedding space (Fig. 7A). The regression models achieved higher-than-chance level decoding accuracies for “across-rat” and “across-region” decoding for the CA1 and for across-rat, across-region, and “across-task” decoding for the SUB (Fig. 7B). To evaluate the similarity of the neural manifold structure, we divided the decoding accuracy of across-rat, across-region, or across-task decoding by that of “self” decoding (*48*). The results revealed partially similar neural manifold structures across rats and regions (Fig. 7C). The structural similarity between the neural manifolds in the T-maze and open-field tasks was higher for the SUB than for the CA1 (Fig. 7C), suggesting that the structure of the neural manifolds in these regions were common across individuals and dependent on the task, particularly in the CA1. Furthermore, common structures between different spatial tasks were observed in the SUB.

### Neural manifold in the SUB better predicts single neuronal activity

**Fig. 8.**
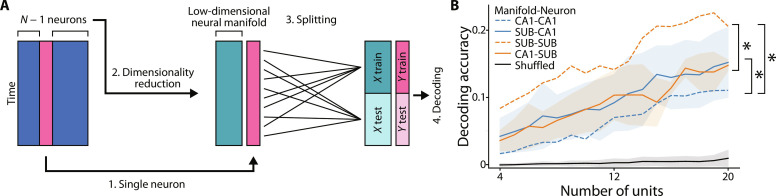
Decoding of single neuronal activities from the CA1 and SUB three-dimensional neural manifolds. (**A**) Schematic illustration of the decoding of single neuronal activities. One neuron was selected as the test data out of the *N* total neurons recorded simultaneously. Then, a neural manifold was constructed using the remaining *N* − 1 neurons to decode the instantaneous firing rate of the selected neuron from this neural manifold. (**B**) Decoding accuracy of single neuronal activity in the CA1 (blue) and SUB (orange). The *x* axis indicates the number of neurons used to construct the neural manifold. Solid lines indicate the decoding of the activity of single neurons in either the CA1 or SUB from the neural manifold in the other region. The dotted lines indicate the decoding of single-neuron activity from the neural manifold in the same region. Labels indicate “neural manifold”–“single neuron.” The black line shows the chance level estimated by the shuffling procedure. Region, *F*_3,334_ = 73.07, *P* < 0.001, two-way ANOVA; *P < 0.05; SUB-SUB versus CA1-CA1, SUB-SUB versus CA1-SUB, and CA1-CA1 versus SUB-CA1; Bonferroni test following two-way ANOVA. *P* < 0.05; SUB-SUB versus SUB-CA1 and CA1-CA1 versus CA1-SUB were omitted.

The higher decoding accuracy with the SUB neural manifold (Figs. 5 and 6) prompted us to investigate whether the SUB neural manifold can better recognize the activity of peer neurons. To test this, we performed the decoding of single-neuron activity from a neural manifold constructed from the remaining neurons (Fig. 8A). We first compared the decoding of single-neuron activity with the neural manifold in the same region (i.e., either the CA1 or SUB) and found that the manifold in the SUB had higher decoding accuracy than that in the CA1 (orange and blue dashed lines in Fig. 8B). Next, we compared the decoding of single-neuron activity from the CA1 and SUB manifolds and found that, regardless of whether the single cells to be decoded were in the CA1 or the SUB, the decoding accuracy was higher when decoding was performed using the SUB manifold (comparison of dashed and solid lines with the same colors in Fig. 8B). These results suggest that the SUB manifold better integrates the activity of peer SUB neurons and upstream CA1 neurons than the CA1 manifold, which potentially contributes to the higher decoding accuracy of behavioral variables.

### Population dynamics during ripples in post-task sleep and its association with awake neural manifold

The CA1 activity during post-task sleep is involved in memory consolidation (*58*). Particular focus has been placed on the replay of population activity during hippocampal SWRs (*58*, *59*). However, the activity in the SUB during SWRs has received limited attention. Therefore, we explored the association between neuronal population activity during wakefulness and post-task sleep ripples in the SUB and CA1.

**Fig. 9.**
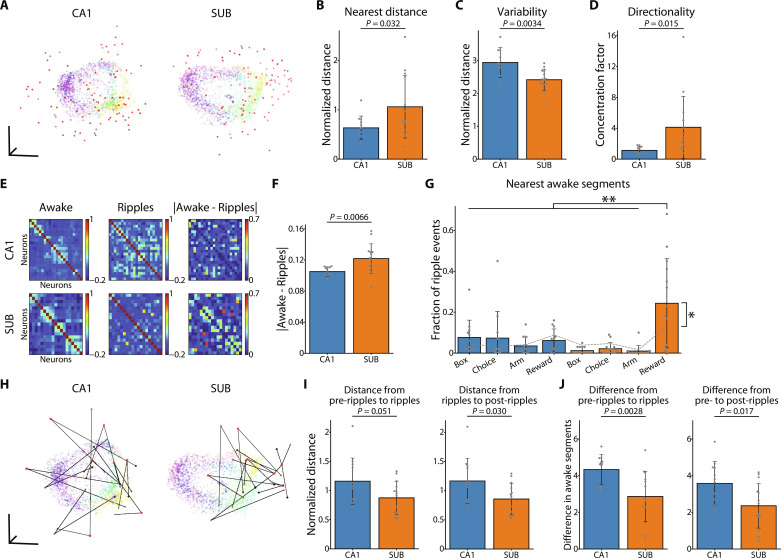
Population dynamics during ripples in post-task sleep. (**A**) Low-dimensional neural manifolds during wakefulness and ripples in the CA1 (left) and SUB (right) visualized using Isomap in a three-dimensional space. Red dots indicate population activity for each ripple event. (**B**) First three nearest neighbor distances from ripples to the awake manifold in the CA1 and SUB. *P* = 0.032, Welch’s *t* test. (**C**) Average of all pairwise distances of ripple events in the CA1 and SUB. *P* = 0.0034, Welch’s *t* test. (**D**) Concentration factors indicating the directionality of ripple points from the awake manifold. *P* = 0.015, Welch’s *t* test. (**E**) Awake (left) and ripple (middle) pairwise correlations and absolute values of differences (right) in the CA1 (top) and SUB (bottom). (**F**) Absolute values of differences in pairwise correlations during wakefulness and ripples. *P* = 0.0066, Welch’s *t* test. (**G**) Nearest neighbor segments from ripples to awake manifold, especially box (first), choice (second), arm (third), and reward (fourth) in the CA1 (blue) and SUB (orange). The gray dashed line shows the null obtained by randomly scattering ripple points. Interaction, *F*_3,96_ = 8.65, *P* < 0.001, two-way ANOVA; **P < 0.005, Bonferroni test following two-way ANOVA; *P = 0.039, paired *t* test. (**H**) Low-dimensional neural manifolds during wakefulness and peri-ripple events (pre-ripple, ripple, and post-ripple) visualized in three-dimensional space using Isomap. Gray, red, and black indicate pre-ripple, ripple, and post-ripple periods, respectively, and black lines indicate the trajectory of each series of events. (**I**) Normalized Euclidean distances between pre-ripple and ripple periods (left) and between ripple and post-ripple periods (right). Left, *P* = 0.051; and right, *P* = 0.030, Welch’s *t* test. (**J**) Distance of nearest awake manifold segments between pre-ripple and ripple periods (left) and between pre-ripple and post-ripple periods (right). Left, *P* = 0.0028; and right, *P* = 0.017, Welch’s *t* test.

To achieve this, we mapped the population activity during the T-maze task and ripples in post-task sleep into the same embedding space (Fig. 9A). The ripple event distribution differed between the CA1 and SUB. Specifically, the embedded ripple activities in the CA1 were more dispersed and located closer to the awake manifold, while those in the SUB were more strongly clustered away from the awake manifold (Fig. 9, B to D).

Thus, we postulated that the patterns of neuronal ensemble recruitment from the awake state to the ripple events in the post-task sleep would differ between regions. To investigate this, we compared the pairwise correlations between neurons during the T-maze task versus during ripples in post-task sleep. The differences in pairwise correlations between the two states were larger in the SUB than in the CA1 (Fig. 9, E and F). This finding suggests that the patterns of neuronal population activity during ripples in post-task sleep versus during wakefulness are distinctly configured in the SUB, in contrast to the CA1, in which the activity during ripples often replays the awake activity patterns (*58*, *59*). Next, we examined the similarity of the ripple activity pattern in post-task sleep to that during the T-maze task. First, we segmented the points on the awake manifold based on the linearized rat position and identified the nearest neighbor segments of the ripple points. We focused on the behaviorally salient segments (i.e., box, choice, arm, and reward areas). The CA1 ripple activities were associated with a wide range of behavioral segments, whereas the SUB ripple activities were concentrated near the reward area (Fig. 9G), with similar proportion to the left and right reward areas (left, 51.0%; and right, 49.0%). The fraction of ripple events associated with the reward area (Fig. 9G) did not correlate with the bimodality coefficient of D2 shape distributions (CA1: *R* = −0.21, *P* = 0.51; and SUB: *R* = 0.52, *P* = 0.057). In addition, this fraction in the SUB did not correlate with the speed decoding accuracy calculated using either all (*R* = 0.28, *P* = 0.33), low-speed (below median, *R* = 0.25, *P* = 0.39), or high-speed (above median, *R* = 0.30, *P* = 0.30) time bins. Therefore, the association with the reward area is a unique feature of post-task SUB ripples independent of behavioral variables.

Last, we investigated the peri-ripple dynamics of population activity in post-task sleep. We embedded the population activity 200 ms before, during, and 200 ms after the ripples in the same space with the population activity during the T-maze task and analyzed the peri-ripple trajectory over time (Fig. 9H). The CA1 tended to change its activity pattern during ripples more extensively than the SUB (Fig. 9I). To investigate the relationship between the peri-ripple dynamics and awake manifold, we identified the nearest neighbor segment for the peri-ripple activity and measured its change over time. The dynamics along awake manifold segments during peri-ripple events were lower in the SUB than in the CA1, suggesting that the SUB stably represents information relevant to specific behaviors (Fig. 9J). In summary, the CA1 exhibited activity patterns closely associated with states of wakefulness, while the SUB displayed distinct activity patterns associated with behaviorally salient events, such as reward acquisition.

## DISCUSSION

We found that SUB population activities during spatial tasks form a low-dimensional neural manifold that encodes diverse navigational information. In both CA1 and SUB, the navigational information could be decoded from the neural manifold. Furthermore, the neural manifolds shared structures across rats and between regions. Given that previous studies have reported diverse tuning of individual SUB neurons (*26*–*34*, *36*, *37*), it has remained unclear whether the SUB forms a neural manifold reflecting the external environment. Moreover, with no correspondence among individual neurons across rats, the across-animal commonality is a finding obtained by the present analysis. Compared with the CA1 manifold, the SUB manifold was characteristic in several aspects: its higher dimensionality, structural similarity to the bent figure-8 shape, shared structure between tasks, and higher decoding accuracy for all types of information examined. During post-task ripples, the population dynamics in the CA1 showed an on/near-manifold, replay-like activity, whereas that in the SUB exhibited off-manifold activity anchored to behaviorally salient information. Collectively, the population activity in the CA1 and SUB distinctly encoded information into neural manifolds. We argue that the SUB acts as a common and efficient carrier of navigational information during tasks and reconfigures the behaviorally salient information into a distinct representation during post-task ripple events.

The activity of dozens of neurons in the CA1 and SUB was represented in approximately three and five dimensions, respectively, during the T-maze task; the higher dimensionality in the SUB was maintained in the open-field task. This finding suggests that, although we identified three types of information (i.e., position, speed, and path) represented on the manifold, the SUB can effectively encode at least two more unknown variables. Thus, the high dimensionality suggests that the SUB integrates multiple types of navigational/nonnavigational information. The full extent of information encoded in the SUB remains to be determined. The information for time (*60*–*62*), object (*63*), border (*33*, *64*, *65*), head direction (*3*, *66*), and other cognitive variables distributed in the CA1 and medial/lateral entorhinal neurons, which directly project to the SUB, would be candidates. The high dimensionality in the SUB as a neuronal population may be closely related to the conjunctive representation of multiple variables in single neurons. The SUB has abundant conjunctive neurons that enable efficient information coding (*32*).

Analyses using persistent homology and D2 shape distribution revealed that the CA1 and SUB neural manifolds had a structure homoeomorphic to the external space through which the animal explored. Such neural manifolds reflecting the external environment would constitute a cognitive map in the brain. We also found that, unlike the CA1 manifold, the SUB manifold was more similar to the bent than planar figure-8 shape during the T-maze task. With this shape, the distances between points in the left and right arms/reward zones can be small on the manifold, which is likely to reflect more similar single-neuron and population activity at these zones in the SUB than in the CA1. Similar activity may be attributed to the axis-tuned and reward cells (*28*, *29*), both of which would be active irrespective of the left/right arms. In addition, the difference between the task demand-bound schematic representation in the SUB and location-bound spatial representation in the CA1 (*30*) may also account for the distinct geometries of the manifold in the SUB and CA1. In any event, the different manifold structures suggest that the population activities of the SUB and CA1 distinctly represent behaviorally relevant information.

A marked finding was that the neural manifold in the SUB achieved higher decoding accuracy than that in the CA1 for all types of navigational information examined. The SUB has fewer principal neurons than the CA1 in rats (~26% fewer), monkeys (~42% fewer), and humans (~57% fewer) (*67*). Therefore, to completely inherit the CA1 information, the SUB requires denser information coding in rats, and we predict that the coding becomes even denser in monkeys and humans. Previous studies have demonstrated that SUB neurons represent speed and path more accurately than CA1 neurons (*31*, *32*), consistent with the present results obtained from the neural manifolds. However, regarding the position, CA1 and SUB individual neurons contain the equivalent amount of decodable information (*31*), which differs from the present result. Notably, the neural manifold reflects not only the activity of individual neurons but also the activity of neuronal ensembles. The observed higher decoding accuracy for pairwise coactivation in the SUB (Fig. 5F) supports this notion. Moreover, the decoding method in this study did not assume the independence of individual neurons but could extract information from the activity of mutually interacting neurons. These effects of neuronal ensembles potentially contribute to the denser manifold encoding in the SUB. In addition, it has been suggested that, as dimensionality increases, the representational space becomes sparser, and, thus, the decoding accuracy improves when individual neurons have broad tuning curves (*68*). Hence, the wide range of tuning curves and conjunctive representations in the SUB (*31*, *32*) may be the additional reasons for the observed higher decoding accuracy. Previous studies (*27*, *30*–*32*) performed neither the manifold analysis nor information decoding from the manifolds. Our study expands the understanding of information representation from the single-neuron and population-activity levels to the dimension-reduced manifold level.

What would be the functional significance of the higher decoding accuracy in the SUB? By matching the numbers of neurons used for decoding, we found that size-matched neuronal populations contained more decodable information in the SUB than in the CA1. Consistently, the SUB neural manifold better predicted the activity of peer SUB neurons and even CA1 neurons than the CA1 neural manifold (Fig. 8). The SUB contains several populations of projection neurons that send axons to different downstream regions (*15*–*19*). Therefore, the downstream regions must read out information from a limited number of SUB neurons; hence, the dense manifold encoding in the SUB would contribute to efficient information transfer to downstream regions.

The population dynamics during ripples in post-task sleep differed between the CA1 and SUB. The CA1 activity during ripples was closely related to the activity during wakefulness. In contrast, the SUB representation was away from the awake manifold but was anchored to the behaviorally salient, reward locations. This pattern is reminiscent of the off-manifold activity that deviates from existing patterns during learning (*69*, *70*).

The SUB may, therefore, reconfigure the information obtained during wakefulness into a distinct representation during post-task ripples, for instance, by uniquely recruiting reward cells (*29*). In our experimental system, it was difficult to disentangle the representation of reward from that of the location of the reward or cessation of movement, which should be clarified in future studies. While the CA1 facilitates memory consolidation by replaying past experiences during ripples (*58*, *59*), the function of the SUB ripples remains unknown. Our results highlight the differences in ripple-associated population activity between these regions. Further research remains warranted to reveal how this difference impacts memory and behavior.

The ring-shaped manifold of the head direction cells (*44*) and toroidal manifold of the grid cells (*46*) are preserved across different environments and brain states (wakefulness versus sleep). In contrast, the shape of the CA1 and SUB manifolds depended on the task (Fig. 1), and the dimension of the CA1 manifolds depends on the task complexity (*48*). The anatomical connectivity supporting flexible manifolds of the hippocampus and SUB might differ fundamentally from the connectivity of head direction cells (*71*) and grid cells, which support rigid manifolds. The relatively random connectivity of the CA3-CA3 recurrent and CA3-CA1 feed-forward connection (*58*, *72*, *73*) may account for the flexible manifolds in the hippocampus, which are likely crucial for adapting to various environments and life demands. Finding anatomical connectivity supporting the mechanisms of generating low-dimensional neural manifolds remains to be fully investigated to deepen our understanding of neural computations (*74*).

From the viewpoint of applied research, the shared manifold structures across rats, regions, and tasks would help to improve brain-computer interfaces (BCIs). Manifold stability has been applied to BCIs to maintain their performance over time (*75*), and the network state across rats can be extracted (*47*, *48*). Furthermore, decoding across regions enables the extraction of information from one region by measuring the neuronal activity of another region: for instance, extracting CA1 information using the neuronal activity of the SUB. Such extraction will facilitate the development of a less invasive BCI that can interpret multiregion information from the recordings of a single region.

Although not addressed in this study, the following should be pursued in future research: First, while this study analyzed dozens of neurons recorded simultaneously, determining how the neural manifold’s estimated dimension, geometry, and decoding accuracy change as the number of recorded neurons increases is important. Whether the complexity or memory demand of the task affects the dimension of neuronal population activity in a brain region–specific manner should also be determined, along with how the dimension and geometry of neural manifolds are associated with the anatomical connectivity among members of the neuronal population. Further, this study analyzed the data recorded after the animals had fully learned the task. Determining whether the dimensions of population activity change and how the neural manifolds evolve as the animal learns the task would help elucidate the involvement of neural manifolds in learning and memory. In addition, circuit intervention using optogenetics would be useful to elucidate how the neural manifold is generated, maintained, and modified in the hippocampal formation. Last, investigating how the dense manifold encoding of the SUB contributes to efficient information transfer to downstream regions will help delineate neuronal communication across brain regions.

## MATERIALS AND METHODS

Data obtained from a previous study (*31*) were used. Analyses of low-dimensional neural manifolds were not reported in that study. All animal care and use procedures were approved by the Institutional Animal Care and Use Committee of Osaka City University (approval no. 15030) and performed in accordance with the National Institutes of Health Guide for the Care and Use of Laboratory Animals. Detailed experimental procedures have been previously reported (*31*). Below, we detail analytical methods and relevant experimental procedures described in the original publication. Data were analyzed using custom scripts written in Python (version 3.8.5), MATLAB (R2018b), and EZR (version 1.40) (*76*).

### Animals and surgery

Eleven male Long-Evans rats (8.9 to 15.3 weeks old on the day of surgery) were used. Under isoflurane anesthesia, rats were implanted with 256-channel silicon probes of eight shanks, with each shank containing 32 recoding sites (Buzsaki256, A8 × 32-5 mm-35-300-160, A8 × 32-Edge-5 mm-25-200-177, NeuroNexus) above the left dorsal SUB and distal CA1 (center of the eight shanks anteroposterior from the bregma, −5.9 to −6.1 mm; mediolateral from the bregma, 2.7 to 3.3 mm; and dorsoventral from the cortical surface, 2.4 mm, with shanks parallel to the coronal plane). Two stainless steel screws were inserted above the cerebellum as indifferent and ground electrodes. The silicon probes were mounted on a three-dimensional–printed microdrive and gradually lowered to the SUB and CA1 after implantation.

### Data collection

Extracellular electrophysiological data were acquired using a 256-channel multiplexed recording system (KJE-1001, Amplipex, Szeged, Hungary). Signals were amplified using a preamplifier module (HS-10, Amplipex) and acquired at 20 kHz with a 16-bit resolution. An overhead camera (c930e, Logicool) was used to track the animal’s position by monitoring two light-emitting diodes (green and red, 5-cm separation) mounted on a head-stage at a sampling rate of ~30 Hz. One pixel in the camera corresponded to 0.52 cm for the open-field and alternating T-maze tasks. LED locations were extracted and resampled to 39.0625 Hz (i.e., 25.6-ms intervals). Spike sorting was first performed automatically using Kilosort (https://github.com/cortex-lab/KiloSort and https://github.com/MouseLand/Kilosort2); thereafter, clusters were manually curated using the Phy graphical user interface (https://github.com/cortex-lab/phy). We used units that met all the following criteria for further analysis: isolation distance (*77*) of >20, interspike interval index (*31*) of <0.2, trough-to-peak amplitude of >50 μV, and overall mean firing rate of >0.1 Hz.

### Behavioral procedures

We trained the rats daily for 7 to 9 days before surgery and 4 to 8 days after surgery on four water-rewarded spatial tasks: open-field, linear-track, alternating T-maze, and zigzag maze tasks. Recording sessions were conducted for two to three consecutive days in which the four behavioral tasks lasted 20 min each with 40- to 80-min rest sessions before, between, and after the tasks per day. We only analyzed the open-field and alternating T-maze tasks. During the behavioral experiments, the rats were deprived of water to maintain ~90% of their free-feeding body weight. All behavioral experiments were performed during the light period of a 12-hour light/dark cycle. In the open-field task, rats freely foraged to obtain randomly scattered water drops in a black square arena (118 cm by 118 cm, depth of 40 cm, A4 size white cue card on one wall). For the alternating T-maze task, we used a square arena (118 cm by 118 cm, depth of 40 cm) consisting of a start box (30 cm by 10 cm), stem (98 cm by 10 cm), and left/right arms (10-cm width). Rats were enclosed for ~8 s in the start box before running through the stem and had to select the alternative arm from that chosen in the previous trial to obtain a water reward at the end of the arm.

### Histology

After making electrical lesions for the localization of silicone probes, rats were transcardially perfused with 0.9% saline, followed by 4% paraformaldehyde in 0.1 M phosphate buffer. Rat brains were postfixed overnight in the same fixative at 4°C and sectioned coronally at a thickness of 50 μm using a vibratome (VT1200S, Leica, Wetzlar, Germany). Sections were stained with 4′,6-diamidino-2-phenylindole (0.5 μg/ml; D1306, Thermo Fisher Scientific, Waltham, MA, USA) and fluorescent Nissl (1:200, N21482, Thermo Fisher Scientific) to identify the recoding sites based on the electrical-lesion locations.

### Cell classification

Putative principal neurons were classified according to the spike width and overall mean firing rate. Putative CA1 principal neurons were defined as units with >0.4-ms spike width and <10-Hz mean firing rate, while putative SUB principal neurons were defined as units with >0.4-ms spike width. We did not set a threshold for the firing rate for the SUB principal neurons, as these cells can exhibit high firing rates (*26*, *31*). We validated the above classification criteria by detecting putative monosynaptic connections using cross-correlograms of the spike trains of two neurons (*31*). In total, 315 CA1 principal neurons and 319 SUB principal neurons were identified and analyzed.

### Preprocessing

For subsequent analyses, we used sessions including ≥10 simultaneously recorded principal neurons with a mean firing rate of >0.1 Hz in each region for each task. We calculated the instantaneous firing rate of each neuron using 512-ms bins unless otherwise stated. To comprehensively capture the population dynamics during the behavioral tasks, we used the entire 20-min task periods for analyses including both theta and non-theta epochs. The construction of the neural manifolds was performed using the square root of the instantaneous firing rate to stabilize the variance (*44*) unless otherwise stated. Rat positions were obtained every 512 ms by extracting a bin in every 20 bins from the 25.6-ms interval position data. The instantaneous *X* and *Y* positions were smoothed separately with a Gaussian filter (σ = 1 bin). The instantaneous running speed was calculated by dividing the distance between the smoothed positions in adjacent time bins by bin size (512 ms) and smoothed with a Gaussian filter (σ = 1 bin). In Fig. 6 (C and D), rat positions were obtained by modifying the number of bins extracted from the 25.6-ms interval position data such that the bin sizes for computing the speed and neural manifold were the same.

### Dimensionality estimation

The time series of neuronal activity can be described as a point cloud in a high-dimensional space with dimensions equivalent to the number of simultaneously recorded neurons. Single points in space represent population activity at the corresponding time points. We estimated the latent dimensionality of population activity, consisting of the instantaneous firing rate of each neuron, embedded in a high-dimensional space using the GP algorithm (*47*, *48*, *53*). The GP algorithm estimates dimensionality using a function of the correlation integral *C*(*r*), which represents the proportion of data point pairs whose constituent points are within distance *r* of each other. The *C*(*r*) was obtained by placing a hypersphere of radius *r* centered at a point and counting the points contained in the hypersphere as follows

$$C\left(r\right)=\frac{1}{N_{\text{pair}}}\sum_{\begin{array}{c} i,j=1\\ i<j \end{array}}^{N}H\left(r-\left\|x_{i}-x_{j}\right\|\right)$$ (1)

where *N* is the number of points, *N*_pair_ is the number of combinations, ***x****_i_* is the vector pointing from the origin to the *i*th point, and *H* is the Heaviside function. The following relationship exists across *C*(*r*), *r*, and the latent dimension α

$$C\left(r\right)\propto r^{\alpha }$$ (2)

Thus, the slope of the log-log plot of *C*(*r*) as a function of *r* corresponds to the dimensionality. We calculated the slope with the least squares method using points with *C*(*r*) values in the range of 20th to 80th percentile to estimate α. To estimate the latent dimensionality of CA1 and SUB population activity, 23.23 ± 12.34 CA1 and 18.80 ± 5.95 SUB principal cells were used for the T-maze task, and 26.50 ± 12.29 CA1 and 18.80 ± 5.95 SUB principal cells were used for the open-field task.

### Dimensionality reduction

To visualize the geometry of high-dimensional point clouds of population activity, we used Isomap, a nonlinear dimensionality reduction method (*44*, *78*) and embedded population activity, consisting of the square root of the instantaneous firing rate with ≥10 dimensions, corresponding to the number of simultaneously recorded neurons, into three dimensions. We used the Gaussian-filtered (σ = 2-time bins) square root of instantaneous firing rates for the analyses in Figs. 1 (F and G), 2, 3, and 9 (A and H) and unsmoothed square root of instantaneous firing rates for the other analyses. The Isomap procedure was performed using the Python package scikit-learn (version 0.23.2) with default settings except for the hyperparameter; the number of neighbors to consider for each point was set to 20.

### Persistent homology

We computed persistent homology to characterize the topological geometry of neural manifolds embedded in a three-dimensional space. We used the Python package Ripser (https://github.com/ctralie/ripser) (*44*, *46*, *54*, *55*) with default settings, except for the hyperparameter “maxdim” that was set to 2. For each point, we set up a sphere whose radius is the first percentile of the distribution of the pairwise distances of all point combinations in the three-dimensional embedding space and excluded points whose number of points in the sphere was below the 20th percentile of neighborhood point distribution (*44*). We considered a sphere centered at every point in the three-dimensional embedding space and gradually increased its radius. At a certain radius, a hole surrounded by these spheres can be generated; as the radius is further increased, the generated hole disappears. The lifetime between the hole’s appearance and disappearance indicates its robustness. Thus, robust and noisy structures have long and short lifetimes, respectively. We used shuffled data to statistically distinguish between robust and noisy geometric structures. The shuffled data were generated by a circular shift of the spike trains of each neuron with uniformly distributed random intervals between 0 and the session length, independent of other neurons. The shuffled data were processed in the same manner as the unshuffled data. We obtained a null distribution by repeating this procedure 50 times, where at least 100 holes for each dimension were generated per procedure. We defined robust holes as geometric structures with lifetimes exceeding the 99.9th percentile of the null distribution. In topology, holes with 0, 1, and 2 dimensions represent components, rings, and cavities, respectively (*79*).

### D2 shape distribution

We characterized the shape signatures of the low-dimensional neural manifold in a three-dimensional embedding space based on the D2 shape distribution (*56*). As in the analysis of persistent homology, points whose neighborhood point density was lower than the 20th percentile of the neighborhood point density distribution were excluded. We computed the distance between two points for all combinations and normalized the distances such that the minimum value was zero and the maximum value was one. We constructed a histogram of distances with 1200 bins. The resulting histogram was smoothed with a Gaussian filter (σ = 10 bins) and used as shape signatures. To compare the shape signatures between the two point clouds, we defined “dissimilarity” as the sum of the absolute value of the probability difference between the data and model for each bin. “Sphere” was created by distributing the point clouds uniformly on a sphere. “Figure-8 shape” was created with two donut shapes with the same radius touching in a plane, and “Bent figure-8 shape” was created with two donut shapes touching at an angle of 90°. We quantified the bimodality of the shape signatures using bimodality coefficient *b*, defined as

$$b=\frac{g^{2}+1}{k+\frac{3{\left(n-1\right)}^{2}}{\left(n-2\right)\left(n-3\right)}}$$

where *n*, *g*, and *k* are the number of samples, skewness, and excess kurtosis, respectively (*80*).

We calculated PVs as the firing rates of a neuronal population at each linearized position for the left and right trials separately and then calculated the PV correlation as the correlation coefficient of PVs between the left and right trials (Fig. 3C).

### Decoding position and speed

We used GPR to predict the position and speed from coordinates on a low-dimensional neural manifold constructed from the square root of unsmoothed instantaneous firing rates (*48*, *57*). GPR is a regression method characterized by nonlinearity and Bayesian estimation. GPR was performed using the Python package scikit-learn (version 0.23.2) with default settings except for the following hyperparameters: “normalize_y” = True and “kernel” = Constant Kernel * RBF + White Kernel. We predicted position and speed by performing twofold cross-validation, in which time bins in each session were randomly sorted into two groups with 50% of the bins; each group was used as test data, and the remaining group was used as training data to obtain the predicted position and speed, and the decoding accuracies were averaged across groups. The decoding accuracy was defined as the coefficient of determination (*R*^2^) between the predicted and observed values as follows

$$R^{2}=1-\frac{\sum_{i=1}^{n}{\left(y_{i}-{\overset{´}{y}}_{i}\right)}^{2}}{\sum_{i=1}^{n}{\left(y_{i}-\bar{y}\right)}^{2}}$$

where *n* is the number of time bins, *y_i_* is the *i*th value of the observed variable, *ý_i_* is the *i*th value of the predicted variable, and $\bar{y}$ is the mean value of the observed variable. Notably, the decoding accuracy of the position was calculated by decoding the *X* and *Y* positions separately and averaging the two decoding accuracy values. For position decoding, we also performed twofold cross-validation, in which each session was divided into the first and second halves; each half was used as test data, and the remaining half was used as training data. Then, the *R*^2^ calculated for each half was averaged across halves to obtain a decoding accuracy for that session; the SUB showed higher decoding accuracy than the CA1 when the number of units constructing the neural manifolds was matched (10 units) (CA1, 0.13 ± 0.078; and SUB, 0.25 ± 0.11; *P* = 0.010, Welch’s *t* test). We compared the decoding accuracy of the neural manifolds constructed from the same number of neurons. To obtain the decoding accuracy, we randomly selected a given number of neurons from the simultaneously recorded neurons independently 50 times, performed the decoding analysis described above, and averaged across the repetition. To investigate the effect of the higher mean firing rates in the SUB than in the CA1 (*26*, *31*), we performed the above-described decoding after equating the mean firing rates of SUB and CA1 neurons by randomly removing spikes from SUB neurons. The chance level of the decoding accuracy was estimated using a shuffling procedure repeated 20 times. The shuffled data were generated by a circular shift of the spike trains of each neuron with a uniformly distributed random interval between 0 and the session length independent of other neurons. Thereafter, the shuffled data were processed in the same manner as the unshuffled data.

### Decoding path

We performed Gaussian process classification (GPC) to predict the next choice (left/right arms) using the neural manifold coordinates constructed from the square root of unsmoothed instantaneous firing rates during the stay in the start box at the beginning of each correct trial of the T-maze task. GPC was performed using the Python package scikit-learn (version 0.23.2) with default settings, except for the following hyperparameter: “kernel” = Constant Kernel * RBF + White Kernel. Using leave-one-out cross-validation, we calculated the choice probability for each time bin and averaged it across time bins for each trial; a turn direction with the resultant higher choice probability was assigned as a predicted choice for that trial. To compare the accuracy of path decoding with the neural manifolds of the CA1 versus the SUB, we randomly and independently selected the same number of CA1 and SUB neurons five times to obtain the averaged decoding accuracy for a given number of neurons.

To predict the choice from pairwise coactivity, we calculated the covariance of the square root of the instantaneous firing rate in the start box of each correct trial for all combinations of neurons. GPC with leave-one-out cross-validation was used to predict the choice from the obtained covariances. To compare the CA1 versus SUB decoding accuracies, we randomly selected the same number of CA1 and SUB neurons 50 times to obtain the averaged decoding accuracy for a given number of neurons.

### Decoding across rats, regions, and tasks

We performed position prediction to determine whether structures of the neural manifold are similar across rats, brain regions, and tasks. Only sessions with ≥0.2 decoding accuracies for the position were included. First, we trained a regression model using the neural manifold coordinates in a three-dimensional space and the position of one rat (“rat A”) using GPR. Subsequently, we applied this regression model to the dataset of another rat (“rat B”) to predict the position from the coordinates on the neural manifold after aligning it with optimal rotation parameters in a three-dimensional embedding space. To this end, we searched for the rotation parameters that predicted the position of rat B using randomly selected data (50% of the total data) with the highest accuracy by systematically rotating the relevant neural manifold of rat B in three dimensions with a special orthogonal group of degree 3 (*48*). We used the optimized rotation parameters to predict the position of rat B from the remaining 50% of the data from the rat B (*48*). For each rat B, the best decoding accuracy obtained using a regression model trained on other rats (as rat A) was assigned as the decoding accuracy of that rat. For across-rat decoding, the data from the same brain regions (CA1 or SUB) during the T-maze task were used for regression model training and decoding. For across-region decoding, neural manifolds from different brain regions during the T-maze task were used for regression model training and decoding. For across-task decoding, neural manifolds from the same brain region were used for regression model training and decoding; the data during the open-field task were used to train the regression model, and the data during the T-maze task were used for decoding.

The chance level of decoding accuracy was estimated using a shuffling procedure. First, we shuffled spike trains of the training dataset by a circular shift of the spike trains of each neuron with a uniformly distributed random interval from 0 to the session length, independent of other neurons and then trained the regression model using the shuffled data in the same manner as that for the unshuffled data. Subsequently, we applied this regression model to the unshuffled dataset of another rat (rat B) in the same manner as that for across-rat and across-region decoding. We repeated the above shuffling, model training, and decoding procedures 100 times for each “rat A–rat B” pair. For each rat (as rat B), we determined the best decoding accuracy achieved by a regression model trained on the shuffled dataset of other rats (as rat A). This best decoding accuracy was assigned as the decoding accuracy of that rat for the shuffling method.

We calculated structural similarity by dividing the decoding accuracy of across-rat, across-region, or across-task decoding by that of self decoding. The self decoding was performed as described in the “Decoding position and speed” section.

### Decoding of single-neuron activity

First, we excluded silent neurons (average firing rate of <0.001 Hz) during the T-maze performance and only considered eight sessions in which at least 10 principal neurons from both the CA1 and SUB were recorded simultaneously. We then randomly selected a neuron as the test data from *N* simultaneously recorded neurons and used the remaining *N* − 1 neurons to construct the neural manifold. The instantaneous firing rate of the selected neuron was smoothed using a Gaussian filter (σ = 1 time bin). Similar to the method described in the “Decoding position and speed” section, we decoded the smoothed instantaneous firing rate of the selected neuron from the neural manifold generated by *N* − 1 neurons. To compare the CA1 and SUB decoding accuracies with the same number of neurons, we randomly selected a given number of neurons from *N* − 1 neurons independently twice, performed the decoding analysis described above, and averaged across the repetition. The chance level of the decoding accuracy was estimated using a shuffling procedure repeated 10 times. We repeated the above process *N* times and averaged the resultant decoding accuracy. The shuffled data were generated by circularly shifting the spike trains of *N* − 1 neurons with uniformly distributed random intervals from 0 to the session length, independent of the other neurons. Thereafter, the shuffled data were processed in the same manner as the unshuffled data to obtain the decoding accuracy of the shuffled data.

### Ripple detection

Ripple activity was examined during post-task slow-wave sleep. As the frequency and duration of ripple events are comparable between the SUB and CA1 (*81*), we adopted the ripple event detection method (*58*) to detect ripples in the SUB. We obtained the ripple-band local field potential (LFP) signal from the center of the SUB cell layer by applying band-pass filtering (140 to 230 Hz). To calculate the normalized ripple power, we used the *z*-scored moving average of the square of the ripple-band LFP signal with a window size of 11 samples. We identified candidate ripple events as periods with a normalized ripple power of >3. We combined candidate events that occurred within 30 ms of each other into a single event, discarded those with a low peak normalized ripple power (<7) and duration that was too short (<15 ms) or too long (>300 ms), and considered the remaining as ripple events. The time of the negative peak of the band-pass–filtered LFP signal of each ripple event was regarded as the ripple peak time. In subsequent analyses, we only included sessions with >100 ripples during post-task slow-wave sleep.

### Pairwise correlations

We calculated pairwise correlations based on the instantaneous firing rates of neurons in the 512-ms time window during wakefulness and in the 76.8-ms time window centered on the ripple peaks in post-task sleep. During wakefulness, we used all time points during task execution, while, during ripples, we randomly selected 100 events for analysis. Neurons with no firing during wakefulness or ripple were excluded.

### Population activity during ripples in post-task sleep

We randomly selected 100 ripple events in post-task sleep and calculated instantaneous firing rates using 76.8-ms bins centered on the ripple peak. Instantaneous firing rates during ripples (76.8-ms bins) and wakefulness (512-ms bins) were square-rooted to stabilize variance and embedded in the same three-dimensional space using Isomap. For visualization (Fig. 9A), we smoothed the square root of instantaneous firing rates during wakefulness with a Gaussian filter (σ = 1024 ms). The coordinate values were *z*-scored. The first three nearest neighbor distances were calculated as the average of the three smallest distances from each activity point during ripples to that during wakefulness. Variability was assessed by calculating all pairwise distances of the activity during ripples and averaging them. Directionality was obtained as follows

$$K=\frac{\bar{R}\left(p-{\bar{R}}^{2}\right)}{1-{\bar{R}}^{2}}$$

where $\bar{R}$ is the mean resultant length of the unit vector from the center of mass of the awake manifold to each ripple point and *p* is the dimension (*p* = 3 in this study) (*82*). For the nearest neighbor segment analysis, we divided the T-maze into 20 equally sized segments along the animal’s path for right and left turn trials separately and identified the center of mass of the manifold points corresponding to each segment. For each ripple event, a segment with a center of mass nearest to the ripple in the manifold was assigned as the nearest neighbor segment, and the fraction of ripple points was quantified for salient positions (i.e., box, choice, arm, and reward positions) without distinguishing between left and right trials.

### Dynamics of peri-ripple population activity during post-task sleep

For each post-task sleep session, we randomly selected 30 ripple events without replacement. For each ripple event, we calculated instantaneous firing rates during, before, and after ripple events using 76.8-ms bins centered on the ripple peak, 200 ms before the ripple peak, and 200 ms after the ripple peak, respectively. Instantaneous firing rates during, before, and after ripples (76.8-ms bins) and wakefulness (512-ms bins) were square-rooted to stabilize variance and embedded in the same three-dimensional space using Isomap. The coordinate values were *z*-scored. For visualization (Fig. 9H), we smoothed the square root of instantaneous firing rates during wakefulness with a Gaussian filter (σ = 1024 ms) and displayed only 10 peri-ripple events. To quantify changes in the population activity around a given ripple event, we calculated the Euclidean distances of the peri-ripple event in the embedding space. To quantify transitions of the nearest neighbor segments of peri-ripple activities (pre-ripple, ripple, and post-ripple), we assigned the nearest neighbor segment to each period and calculated the difference between the nearest neighbor segments. We repeated the random selection of 30 ripple events after the replacement of the previously selected 30 ripple events 10 times to include the most ripple events in the analysis and reported the mean of the resultant distance values.

### Statistical analyses

Statistical analyses were performed using Python and EZR (*76*). Data are expressed as the means ± SD. We used the paired *t* test for within-group comparisons and Welch’s *t* test for between-group comparisons. Paired *t* tests with Bonferroni correction following one-way repeated-measures ANOVA were used to evaluate the differences among multiple paired groups. The Bonferroni test, following two-way ANOVA, was used for multiple comparisons. All tests were two-sided, and *P* values of <0.05 indicated statistical significance.
