# Supplementary material for: Distinct manifold encoding of navigational information in the subiculum and hippocampus
Source: Sci Adv. 2024 Jan 31;10(5):eadi4471. doi: 10.1126/sciadv.adi4471 (PMC10830115; doi:10.1126/sciadv.adi4471)
Supplement: Supplementary file 1 — Legend for data S1 [file sciadv.adi4471_sm.pdf]

Science Advances
AAAS

Supplementary Materials for

Distinct manifold encoding of navigational information in the subiculum and hippocampus

Shinya Nakai *et al.*

Corresponding author: Kenji Mizuseki, mizuseki.kenji@omu.ac.jp;
Takuma Kitanishi, tkitanishi@g.ecc.u-tokyo.ac.jp

Sci. Adv. **10**, eadi4471 (2024)
DOI: 10.1126/sciadv.adi4471

The PDF file includes:

Legend for data S1

Other Supplementary Materials for this manuscript include the following:

Data S1

Data S1. Source data for Figures 1–9. Individual CSV files contain source data for each figure panel as follows.

fig1b_sourcedata.csv (source data for Fig. 1B)
fig1e_sourcedata.csv (source data for Fig. 1E)
fig1f_sourcedata.csv (source data for Fig. 1F)
fig1g_sourcedata.csv (source data for Fig. 1G)
fig2a_sourcedata.csv (source data for Fig. 2A)
fig2b_sourcedata.csv (source data for Fig. 2B)
fig3a_sourcedata.csv (source data for Fig. 3A)
fig3b_sourcedata.csv (source data for Fig. 3B)
fig3c_sourcedata.csv (source data for Fig. 3C)
fig4a_sourcedata.csv (source data for Fig. 4A)
fig4b_left_sourcedata.csv (source data for Fig. 4B, left)
fig4b_right_sourcedata.csv (source data for Fig. 4B, right)
fig4c_sourcedata.csv (source data for Fig. 4C)
fig4d_left_sourcedata.csv (source data for Fig. 4D, left)
fig4d_right_sourcedata.csv (source data for Fig. 4D, right)
fig4e_sourcedata.csv (source data for Fig. 4E)
fig4f_sourcedata.csv (source data for Fig. 4F)
fig4g_sourcedata.csv (source data for Fig. 4G)
fig4h_sourcedata.csv (source data for Fig. 4H)
fig5a_sourcedata.csv (source data for Fig. 5A)
fig5b_sourcedata.csv (source data for Fig. 5B)
fig5c_sourcedata.csv (source data for Fig. 5C)
fig5_inset_sourcedata.csv (source data for Fig. 5D, inset)
fig5d_sourcedata.csv (source data for Fig. 5D)
fig5e_sourcedata.csv (source data for Fig. 5E)
fig5f_sourcedata.csv (source data for Fig. 5F)
fig6a_right_sourcedata.csv (source data for Fig. 6A, right)
fig6a_sourcedata.csv (source data for Fig. 6A)
fig6b_sourcedata.csv (source data for Fig. 6B)
fig6c_right_sourcedata.csv (source data for Fig. 6C, right)
fig6c_sourcedata.csv (source data for Fig. 6C)
fig6d_sourcedata.csv (source data for Fig. 6D)
fig6e_sourcedata.csv (source data for Fig. 6E)
fig6f_sourcedata.csv (source data for Fig. 6F)
fig_7b_sourcedata.csv (source data for Fig. 7B)
fig_7c_sourcedata.csv (source data for Fig. 7C)
fig8b_sourcedata.csv (source data for Fig. 8B)
fig9a_sourcedata.csv (source data for Fig. 9A)
fig9b_sourcedata.csv (source data for Fig. 9B)
fig9c_sourcedata.csv (source data for Fig. 9C)
fig9d_sourcedata.csv (source data for Fig. 9D)
fig9e_sourcedata.csv (source data for Fig. 9E)
fig9f_sourcedata.csv (source data for Fig. 9F)

fig9g_sourcedata.csv (source data for Fig. 9G)
fig9h_sourcedata.csv (source data for Fig. 9H)
fig9i_sourcedata.csv (source data for Fig. 9I)
fig9j_sourcedata.csv (source data for Fig. 9J)
